# A Genome-Wide Linkage and Association Scan Reveals Novel Loci for Hypertension and Blood Pressure Traits

**DOI:** 10.1371/journal.pone.0031489

**Published:** 2012-02-24

**Authors:** Youling Guo, Brian Tomlinson, Tanya Chu, Yu Jing Fang, Hongsheng Gui, Clara S. Tang, Benjamin H. Yip, Stacey S. Cherny, Yoon-Mi Hur, Pak Chung Sham, Tai Hing Lam, Neil G. Thomas

**Affiliations:** 1 The State Key Laboratory of Brain and Cognitive Sciences, Department of Psychiatry, The University of Hong Kong, Hong Kong, China; 2 School of Public Health, The University of Hong Kong, Hong Kong, China; 3 Department of Medicine and Therapeutics, Chinese University of Hong Kong, Hong Kong, China; 4 Mokpo National University, Seoul, South Korea; 5 Unit of Public Health, Epidemiology and Biostatistics, University of Birmingham, Birmingham, United Kingdom; University of Utah, United States of America

## Abstract

Hypertension is caused by the interaction of environmental and genetic factors. The condition which is very common, with about 18% of the adult Hong Kong Chinese population and over 50% of older individuals affected, is responsible for considerable morbidity and mortality. To identify genes influencing hypertension and blood pressure, we conducted a combined linkage and association study using over 500,000 single nucleotide polymorphisms (SNPs) genotyped in 328 individuals comprising 111 hypertensive probands and their siblings. Using a family-based association test, we found an association with SNPs on chromosome 5q31.1 (rs6596140; *P*<9×10^−8^) for hypertension. One candidate gene, *PDC*, was replicated, with rs3817586 on 1q31.1 attaining *P* = 2.5×10^−4^ and 2.9×10^−5^ in the within-family tests for DBP and MAP, respectively. We also identified regions of significant linkage for systolic and diastolic blood pressure on chromosomes 2q22 and 5p13, respectively. Further family-based association analysis of the linkage peak on chromosome 5 yielded a significant association (rs1605685, *P*<7×10^−5^) for DBP. This is the first combined linkage and association study of hypertension and its related quantitative traits with Chinese ancestry. The associations reported here account for the action of common variants whereas the discovery of linkage regions may point to novel targets for rare variant screening.

## Introduction

Hypertension, which affects nearly 27% of the population worldwide [1], is a major cause of cardiovascular morbidity and mortality [2]. The condition is highly heritable and polygenic, caused by the combination of small changes in the expression of many genes, and interaction of these genes with multiple environmental factors [3]. The identification of allelic variation affecting blood pressure in the general population would advance our understanding of blood pressure regulation and may contribute to the development of approaches for prevention and treatment of hypertension in the future.

The high heritability (30–60%) of blood pressure has prompted extensive efforts to dissect its genetic basis [4]. In the 90 s and the early part of the present decade, genome-wide linkage analysis had been one of the main strategies for identifying hypertension susceptibility genes. It involves the genotyping of hundreds or thousands of markers in families with one or more hypertensives. Using this method, linkage studies have found significant or suggestive loci influencing blood pressure as a quantitative trait or hypertension as a qualitative trait [5], [6], [7], [8], [9]. These results indicate that no single genomic region has a uniformly large effect on predisposition to hypertension. Over 100 hypertension-related quantitative trail loci (QTLs) have been demonstrated across the genome; however, the indicated linkage peaks have been too broad and unstable, likely due to insufficient sample size and the highly polygenic nature of the condition, and as a result there have been few replications between populations [10].

Genome-wide association studies (GWAS), using hundreds of thousands of single nucleotide polymorphism (SNP) markers, are the current method of choice for dissecting the genetic basis of complex disease and can provide a potentially more powerful method of identifying the causal variants that underlie susceptibility to common disease, including hypertension, as compared to linkage analysis. From 2007 to 2009, there have been several large-scale GWAS of hypertension. One of them [11] was carried out by the Wellcome Trust Case Control Consortium (WTCCC) and no significant variants were yielded in the initial and replication stage of data analysis. A meta-analysis reported the combined findings of two consortia: the Cohorts for Heart and Aging Research in Genome Epidemiology (CHARGE) Consortium and the Global BPgen Consortium, with very large study samples of European ancestry [12], [13]. Four loci for systolic blood pressure (SBP), six for diastolic blood pressure (DBP) and one for hypertension attained genome-wide significance. Studies from the Global BPgen consortium, followed up by direct genotyping and *in silico* comparison (CHARGE consortium), identified association with SBP or DBP in eight regions. GWAS on young-onset hypertension [14] were carried out in the Han Chinese population of Taiwan and located 4 SNPs with strong association signals. Another GWAS [15] was conducted in the Old Order Amish (a closed founder population of European origin) and identified a novel susceptibility gene, *STK39*.

The present study used sibships recruited in Hong Kong, where one member of the sibship is hypertensive and at least one additional member is normotensive, with as many members of the sibship included in the study as available. We employed the Illumina HumanHap610-Quad Array for genotyping all phenotyped sibship members. This unique use of a family-based design allows testing for both linkage and association for loci influencing hypertension and its related quantitative traits. Here, we present the extensive analyses performed using this high-density SNP data and identify independent and novel genome-wide significant results by both linkage and association analyses.

## Methods

### Ethics Statement

This study was approved by the Institutional Review Board of the University of Hong Kong/Hospital Authority Hong Kong West Cluster, reference UW06-177 T/1202. Consent was obtained from all participants involved in this study with a Participant Information Statement and Consent Form, which was approved by the ethics committee.

### Study samples

The hypertensive subjects were identified in the hypertension and general outpatient clinics of the Prince of Wales Hospital in Hong Kong and referred to the Clinical Pharmacology Studies Unit (CPSU). Hypertensive probands were ascertained, along with as many of their siblings who agreed to participate. Treatment for hypertension was withdrawn to enable the determination of off-treatment blood pressure (BP). Following withdrawal of antihypertensive medication, subjects were monitored weekly to check if their BP did not exceed the upper exclusion limit and to confirm that they were not at risk from severe hypertension. After the four to eight week washout period [16], the sitting SBP and DBP were measured in triplicate after a 10 to 15 minute resting period. Mean arterial pressure (MAP) was calculated from those measures (MAP = [(2× DBP)+SBP]/3). Siblings with SBP >140 mm Hg and/or DBP >90 mmHg were considered hypertensive, with those remaining classified as normotensive. The sample included 328 individuals (143 males, 185 females) comprising 111 sibships ranging in size from 2–8 sibs. The clinical characteristics of study samples are shown in Table 1. The study was approved by the Joint Chinese University of Hong Kong and New Territories East Cluster Clinical Research Ethics Committee, Hong Kong. Signed informed consent was obtained from the subjects.

**Table 1:** Characteristics of the study and population samples.

	Study samples	Population samples
No. of subjects	315	2895
No. of families	111	-
Mean age (years) ± standard deviation	40.3±7.9	45.8±12.9
Sex (proportion male)	0.439	0.488

	patients	healthy siblings	
Hypertension (dichotomous trait)	134	181	-
Mean body mass index(BMI), kg/m^2^	26.9±3.8	24.0±3.4	28.4±3.5
Mean systolic blood pressure (SBP), mm Hg	151.2±13.2	117.6±11.2	119.3±19.8
Mean diastolic blood pressure (DBP), mm Hg	93.8±9.4	70.0±10.5	74.7±10.8
Mean mean arterial pressure (MAP), mm Hg	113.4±11.3	88.9±11.0	89.6±13.0

### Genotyping

Genotyping was performed by deCODE Genetics, Inc, using the Illumina HumanHap 610-Quad BeadChip technology, which enables whole-genome genotyping of 620,901 single nucleotide polymorphisms (SNPs). Image intensities were extracted using Illumina's BeadScan software. Data for the BeadChip were self-normalised using information contained within the array. Allele calling was carried out using Illumina's Genotyping Module version 3.3.7 in BeadStudio version 3.1.3.0.

### Data cleaning

#### Criteria for exclusion of individuals

The first stage of data-cleaning examined the genetic relationships by checking the genome-wide identity by descent (IBD) sharing for all pairs of individuals in the sample after pruning markers in LD (r2>0.25). Based on the pair-wise IBD estimation, 4 individuals distributed in 4 families were recognized to be half-siblings to the probands and recoded as such, and 6 individuals were excluded because of sample contamination or unknown familial relationships. Then, inbreeding coefficients were calculated, which found five individuals with either strong positive or negative inbreeding coefficient estimates, indicating that these individuals have more or fewer homozygous genotypes than one would expect by chance, reflecting potential sample contamination. Gender status was also checked using the X chromosome data, which detected 10 problematic samples for whom the reported sex did not match the estimated sex and were removed since these discrepancies could not be reconciled with our records. After cleaning, 315 individuals within 111 families remained.

#### Criteria for exclusion of SNPs

SNP genotyping quality was evaluated by examining the genotyping call rate (GCR), the minor allele frequency (MAF), and testing for Hardy-Weinberg Equilibrium (HWE). 8400 SNPs with missing rate >5%, 92169 SNPs with MAF<0.01, and 1686 markers which violated HWE (*p*<0.0001) were excluded from analysis. 503,984 SNPs passed quality control (QC) and were used for analysis. The total GCR in remaining individuals was 99.94%, with all chips having a call rate >98%.

### Statistical Analysis

In all linkage and association analyses, age, sex, and BMI were used as covariates for DBP, SBP and MAP values. The standardized values of the residuals obtained were all approximately normally distributed.

#### Family-based association analysis

Univariate family-based association tests were conducted for quantitative traits of DBP, SBP and MAP, and the disease trait of hypertension. Quantitative trait association was performed using the QFAM procedure in PLINK [17], which provides both a total test of association and a within-family test, which is free from bias due to population stratification. The tests employ a simple linear regression of phenotype on genotype and use a permutation procedure to correct for family structure.

For within-family tests, measures of three quantitative traits were each adjusted for age, sex, and BMI. For the total association test, in addition to the above three covariates, in an attempt to correct for population stratification, the first principal component extracted from an EIGENSTRAT [18] analysis was used as an additional covariate. For analysis of the hypertension binary disease trait, the DFAM procedure using the Cochran-Mantel-Haenszel test was employed, which implements a within-family test.

#### Linkage analysis

Linkage was evaluated using MERLIN-REGRESS (version 1.1.2), a regression-based method suitable for selected samples [19]. The method requires specification of population estimates of the trait means and variances. We obtained such estimates from a population-based cohort of Chinese subjects, participating in the Hong Kong Cardiovascular Risk Factor Study [20], shown in Table 1. The linkage analysis also requires specification of trait heritability, and 0.6 was used for all blood pressure traits, as suggested by previous studies [21], [22], [23].

The presence of linkage disequilibrium (LD) among markers violates an underlying assumption of linkage in multipoint linkage approaches. Appropriate handling of marker LD can avoid such false positive evidence [24]. MERLIN has a built-in option of modeling LD (-rsq) by organizing markers into clusters using pre-specified *r* ^2^ equal to 0.25 [25].

We also employed QTDT [26] to conduct a combined test of linkage and association, which can help determine whether a putative QTL locus detected via association is the actual disease-causing locus or whether it is merely in disequilibrium with the trait locus [27], [28].

## Results

### Association analysis

**Figure 1:**
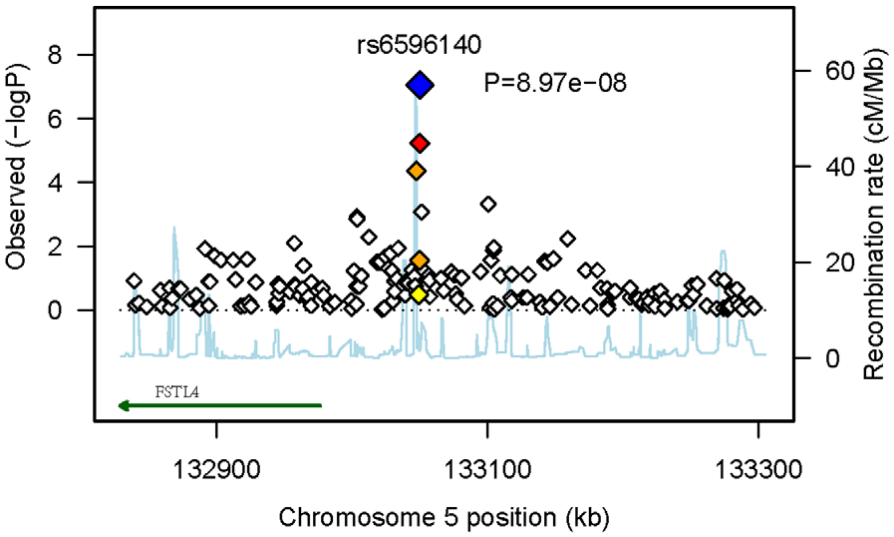
Regional plot of the strongest association for dichotomous hypertension. The plot highlights the statistical strength of the strongest association (rs6596140, *P*<9×10^−8^, blue diamond) and surrounding markers, along with the pair-wise correlations between the surrounding markers and the putative associated variant, indicated by color. All SNPs in the region are plotted with their *p*-values (as –log_10_ values) as a function of genomic position (using NCBI Build 36). Estimated recombination rates (taken from HapMap) are plotted to reflect the local LD structure around the associated SNP and their correlated proxies (bright red indicating highly correlated, faint red indicating weakly correlated). The annotated gene *FSTL4* (taken from UCSC table browser) is more than 70 kb away from the associated SNP.

The whole-genome association scan for hypertension (disease status) and corresponding quantile-quantile (QQ) plot of observed *p*-values against those expected under the null hypothesis are presented in Figure S1. For the dichotomous variable of hypertension, four independent signals were found with *P*≤10^−5^, with one SNP (rs6596140) attaining *P*<10^−7^ (Table S1). The location of this SNP (5q31.1), marked by a strong recombination hotspot, is far away from any annotated genes, and the nearest gene is follistatin-like 4 (*FSTL4)* (>70 kb away) (Figure 1). *FSTL4*, a member of the follistatin gene family of TGF-beta superfamily inhibitors, is widely expressed in neurons, cardiac muscle cells, smooth muscle cells and intestinal epithelium [29]. If the associated SNP is truly associated, it may play a role in regulating this gene, or tagging another SNP having such a role. Associations with SNPs outside of genes is a common phenomenon in genome-wide association studies [30].

Results of the association analysis for the quantitative traits are summarized in Figure S2 and Figure S3. For the within-family test analysis, we observed 10 independent signals with *P*≤10^−5^ for DBP, 1 for SBP and 9 for MAP. For the total association test, we observed 6 independent signals with *P*≤10^−5^ for DBP, 1 for SBP and 3 for MAP. Results of the most significant SNPs are presented for the three quantitative traits in Table S2. The QQ plots of observed *p*-values against those expected under the null hypothesis suggest an excess of associations with DBP and MAP in within-family tests, as compared to a null distribution of no associations, but no distinct deviations for other tests (Figure S4). Population stratification was not a problem in our data, as indicated by a genomic inflation factor λ = 1, based on the median chi-square, for all sets of tests, as expected with use of within-family tests of association or when correcting for stratification as done for the total test of association.

Nine of the SNPs listed in Table S3 are found using all three continuous traits. Because the three traits are correlated (*r*∼0.85–0.90), this is not too surprising. The observation that each SNP shows stronger association with one trait or another could reflect sampling variation or true differences in the underlying biological basis of the blood pressure traits.

For the nine SNPs that were significant in continuous trait analysis, two markers (rs6596140 and rs6596142, *r* ^2^ = 0.84) were associated with hypertension status, with *P*<9×10^−8^ and <6×10^−6^, respectively. Five other markers (rs4463623, rs4434808, rs1387343, rs12930697, rs1550823) had moderate *P* values around .002 to .0004. The other two markers (rs9325113, rs2075514) showed no association.

We also looked for association signals at a subset of our SNPs where there was previous evidence of involvement in hypertension, from both genome-wide association studies [11], [12], [13], [14], [15], [31], [32], [33] and candidate gene studies [34], [35], [36], allowing for a much less stringent multiple testing correction. For SNPs which were not assayed by the Illumina 610-Quad, tag SNPs (*r* ^2^>0.8) were selected instead. In total, this involved examining 101 SNPs. One SNP in *PDC* on 1q31.1 was significantly associated after Bonferroni correction for multiple testing of 101 SNPs, with rs3817586 (tagging rs11812050, *r* ^2^ = 0.95) attaining *P* = 2.5×10^−4^ and 2.9×10^−5^ in the within-family tests for DBP and MAP, respectively. Nonetheless, when examining the QQ plot of these tests (Figure S5), there were only small deviations from what is expected under the null hypothesis, suggesting a lack of power. Phosducin (Pdc), which was identified in retina and brain as a 33-kDa protein and binds to the βγ subunits of heterotrimeric GTP-binding proteins [37], [38], is a potential candidate gene for retinitis pigmentosa [39]. Beetz et al. [35] investigated the role of the G protein regulator Pdc in hypertension and found that Pdc was significantly associated with both wake and stress-response blood pressure phenotypes. It is demonstrated that *PDC* is an important modulator of sympathetic activity and blood pressure and may thus represent a promising target for treatment of stress-dependent hypertension. The family-based association results of all three quantitative traits (DBP, SBP and MAP) for the replicated 101 SNPs from previous studies are presented in Table S4.

### Linkage analysis

Linkage plots from the multipoint analyses using MERLIN-REGRESS are shown in Figure S6 for DBP, SBP and MAP. Linkage analysis using DBP located the highest LOD score on chromosome 5 (LOD = 4.02, p<10^−5^) around 38 cM (1-LOD support interval 34 to 39 cM). The nearest gene is *GDNF*,, glial cell derived neurotrophic factor isoform, which encodes a highly conserved neurotrophic factor and has been found to be associated with the development of schizophrenia [40], [41]. A peak was also seen on chromosome 5 (LOD = 3.85, p<10-5) at around 35 cM, near *AGXT2* which encodes the mitochondrial alanine-glyoxylate aminotransferase. Baker et al. [42] cloned human *AGXT2* and their findings suggest that human hepatocyte mitochondria possess *AGXT2* activity. For the SBP phenotype, the highest peak was found on chromosome 2 (LOD = 3.01, p<10-4) at around 144 cM (130–145 cM), encompassing *ARHGAP15*, or RHO GTPase-activating protein 15. RHO GTPases regulate diverse biologic processes, and their activity is regulated by RHO GTPase-activating proteins (GAPs), such as *ARHGAP15* [43]. Seoh et al. [43] found that the GAP domain of *ARHGAP15* showed specificity toward *RAC1 in vitro*, suggesting that *ARHGAP15* is a regulator of *RAC1*. Suggestive linkage was found for MAP on chromosome 2 (LOD = 2.34, P<.0005) at around 157 cM and chromosome 5 (LOD = 2.31, P<.0006) at around 146 cM. The peak on chromosome 2 encompasses one interesting gene, *GPD2*, which encodes protein to localize to the inner mitochondrial membrane, and also contributes to the genetic liability of type 2 diabetes [44]. An interesting gene within the peak on chromosome 5 is *PPP2R2B*, which encodes a brain-specific regulatory subunit B of protein phosphatase 2. Although the precise role of the subunit encoded by *PPP2R2B* remains to be determined, protein phosphatase 2A (PP2A) has been implicated in a number of cellular functions [45], including cell growth and division, muscle contraction, and gene transcription.

In comparison of the linkage results with other studies in Chinese populations, we found three regions with previous evidence of linkage for hypertension (Figure S7). A linkage peak on chromosome 2q14-q23 for essential hypertension was previously detected in Han Chinese, with a suggestive LOD of 2.24 at 160.52 cM [46]. Fang et al. [47], [48] found linkage to two genes, dopamine D2 receptor (*DRD2*) and Angiotensinogen (*AGT*), with variants contributing an increased risk of hypertension in Chinese subjects. Howerer, none of these regions show strong linkage results in our study.

**Figure 2:**
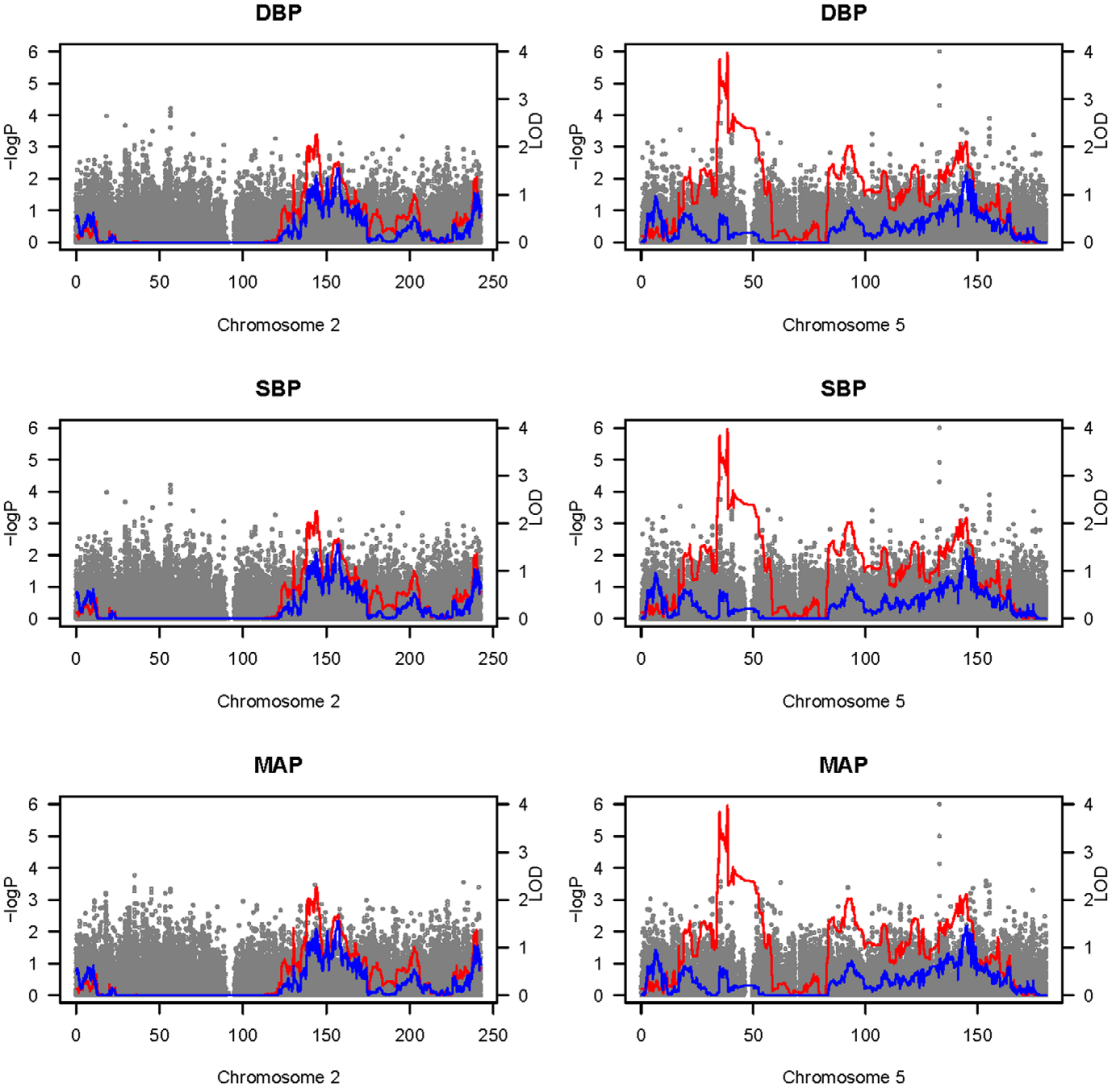
Combined plots for the tests of within-family association and tests of linkage, both in the presence and absence of association. The genome-wide linkage results are shown, with the red lines indicating linkage tests (LOD) in the absence of association, and the blue lines indicating linkage tests (LOD) in the presence of association. The grey dots indicate the within-family association results (-log*P*) in the absence of linkage. All results are shown for DBP, SBP and MAP on chromosomes 2 and 5.

We also performed the simultaneous analysis of both linkage and association for the three quantitative traits. In effect, this allows partialing out the effects of association from the test of linkage. Combined plots for the two chromosomes with strongest linkage peaks (chromosomes 2 and 5) of the tests of within-family association, tests of linkage (when not modeling association), and the combined tests of linkage and association, are presented in Figure 2. When linkage was tested without simultaneous modeling of association, one significant LOD score of 4 for DBP on chromosome 5 was found, along with a suggestive LOD score of 3 for SBP on chromosome 2. By the inclusion of association, the extent of linkage evidence was diminished substantially, with the linkage peaks declining by over 1 LOD score. Nonetheless, since these LOD scores did not decline to zero, it is doubtful that the associated SNPs under these peaks were themselves causal and instead they were likely tagging a causal variant. The strongest peak containing the significant LOD = 4 for DBP on chromosome 5 covered a range of 4 Mb with 808 markers, as defined by the 1 = LOD support interval. Examination of the QQ plots for the 808 SNPs in this region, for both the within- and total-association tests, suggests a clear deviation from that expected under the null hypothesis (Figure S8). The most significant *P* value under the linkage peak was for rs1605685 (*p*<7×10^−5^) for DBP, which was significant after Bonferroni correction for the 808 SNPs under the peak, but no gene has been characterized around this SNP in the literature.

## Discussion

We tested 503,984 SNPs for association with hypertension status and BP traits. Our study is the first report of a combined genome-wide linkage and association scan for these traits in families with Chinese ancestry. The two traits are correlated and heritable, and SBP shows stronger increases with age, with DBP starting to plateau and in some individuals fall at ages above 60–65 years [49]. Some [50] have suggested the study of mean arterial pressure, which increases with advancing age and is highly correlated with SBP and DBP, also showed evidence of heritability. In our GWAS, we chose to examine SBP, DBP and MAP as separate traits, and also carried out an association analysis of hypertension disease status.

We report significant genome-wide linkage as well as an association of common genetic variants with hypertension. Our linkage analysis revealed two novel regions of linkage for DBP, 5p13.1 and 5p13.2, formally achieving genome-wide significance, and also one novel region for SBP, 2q22.3 (LOD = 3.01). Our association analysis has identified a significant variant (rs6596140) for hypertension and also detected effects of relatively common alleles with modest *P* values for quantitative traits. The region of association (Figure 1) is limited to a 3-kb region flanked by strong recombination hotspots, which are surrounded by multiple non-coding sequences but no known genes. *FSTL4*, which is a key modulator in muscle development, was specified as nominally associated with increased risk of stroke in a cardiovascular health study [51]. It is possible that the nearby associated SNP we detected plays a regulatory role for *FSTL4*.

There is limited overlap between the regions of strongest linkage and association. However, further assessment of SNP association under the linkage peak on chromosome 5 did yield a significant variant, rs1605685, after correcting for multiple testing of the 808 SNPs under the peak.

Both linkage and association analysis provide useful, but different forms of information in identifying genetic contributions to complex traits. Linkage analyses are used with family data to find broad genomic regions that contain putative disease loci, while association analyses can identify much smaller regions, either a causal variant or one which is in linkage disequilibrium with such a disease-causing locus. While association can generally detect smaller effects than linkage, association is limited to detecting variants that are either directly assayed or are in strong LD with typed SNP. These are more likely to be common variants, which generally have small effects. In contrast, linkage in families can detect rare variants specific to a subset of families and these rare variants are likely to have larger effects. An advantage of family data in association analyses is to provide a perfect control for population stratification. Our combined linkage and association design brings the best of both worlds to the problem at hand. If significant linkage is detected in the presence of significant association, it suggests that the putative locus is not the functional gene, but rather is a locus in disequilibrium with a trait locus. In contrast, if the linkage signal disappears at a point of significant association, this is suggestive that the associated SNP is, in fact, causal. In addition, all our subjects are Hong Kong Han Chinese, a relatively homogeneous group with regard to genetic background and environmental risk factors. Nevertheless, our findings are limited by relatively small sample size. Furthermore, as prior studies have reported ethnic differences in frequencies of alleles and effects of genes involved in blood pressure traits [52], [53], the novel loci we found may not be generalized to other population groups and await further replication.

## Supporting Information

Figure S1 **Whole genome association scan and QQ plots for dichotomous hypertension.**
(TIF)

Figure S2 **Plots of whole genome association scan results for three quantitative traits using within-family tests.** SNPs from each chromosome are represented by a different color and ordered by physical location.
(TIF)

Figure S3 **Plots of whole genome association scan results for three quantitative traits using the total association test.** SNPs from each chromosome are represented by a different color and ordered by physical location.
(TIF)

Figure S4 **QQ plots of** ***P*** **-values observed vs expected under the null hypothesis, for the three quantitative traits (DBP, SBP and MAP), obtained from tests of within-family and total association.**
(TIF)

Figure S5 **QQ plots for previous reported SNPs by eight genome-wide scan studies as well as candidate gene studies in our association analysis of within-family and total tests for DBP, SBP and MAP.**
(TIF)

Figure S6 **Results of the genome-wide linkage analysis are illustrated for DBP, SBP and MAP, respectively.** The multipoint LOD scores are shown on the *y*-axis plotted against the chromosomal position on the x-axis.
(TIF)

Figure S7 **Three regions (** ***AGT*** **, 2q14-q23 and** ***DRD2*** **) with evidence of linkage are shown in the results of linkage analysis as illustrated for DBP, SBP and MAP, respectively.** The multipoint LOD scores are shown on the *y*-axis plotted against the chromosomal position on the x-axis.
(TIF)

Figure S8 **QQ plots of the Merlin-Regress peak findings in within-family association analysis for DBP.**
(TIF)

Table S1 **Strongest associations obtained for dichotomous hypertensive/normotensive disease status, sorted by P value.**
(PDF)

Table S2 **Strongest associations obtained from both within and total tests for DBP, SBP and MAP, sorted by P value.**
(PDF)

Table S3 **Relationship of SNPs at 9 significant loci to three blood pressure traits.**
(PDF)

Table S4 **Family-based association results for all three quantitative traits (DBP, SBP and MAP) for the 101 SNPs previously reported.**
(XLSX)
