# Correction: A Genome-Wide Linkage and Association Scan Reveals Novel Loci for Hypertension and Blood Pressure Traits

**DOI:** 10.1371/annotation/4415f88f-ab10-44dd-8ba9-1a57ade740c1

**Published:** 2012-06-01

**Authors:** Youling Guo, Brian Tomlinson, Tanya Chu, Yu Jing Fang, Hongsheng Gui, Clara S. Tang, Benjamin H. Yip, Stacey S. Cherny, Yoon-Mi Hur, Pak Chung Sham, Tai Hing Lam, Neil G. Thomas

The current address for author Yu Jing Fang was not indicated. It is: Department of Colorectal Surgery, State Key Laboratory of Oncology in South China, Cancer Center, Sun Yatsen University, Guangzhou, China.

