# Supplementary figures and images for: A Genome-Wide Linkage and Association Scan Reveals Novel Loci for Hypertension and Blood Pressure Traits
Source: PLoS One. 2012 Feb 24;7(2):e31489. doi: 10.1371/journal.pone.0031489 (PMC3286457; doi:10.1371/journal.pone.0031489)

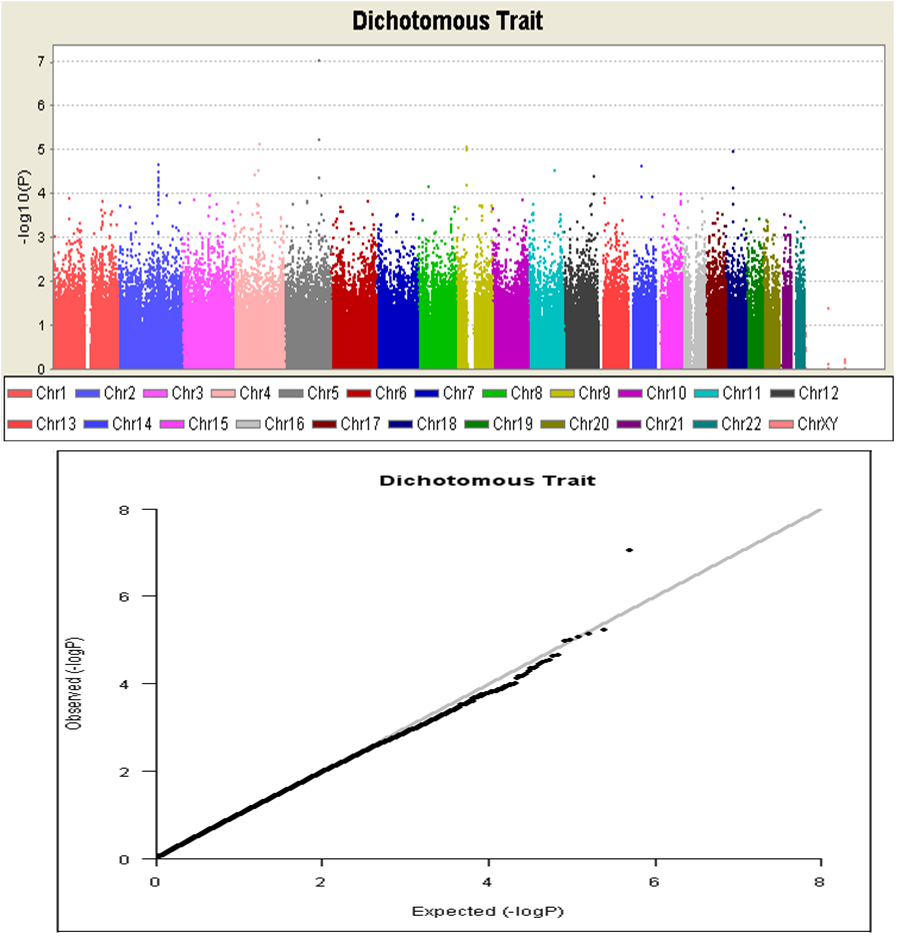

Supplement: Figure S1 — Whole genome association scan and QQ plots for dichotomous hypertension. (TIF) [file pone.0031489.s001.tif]

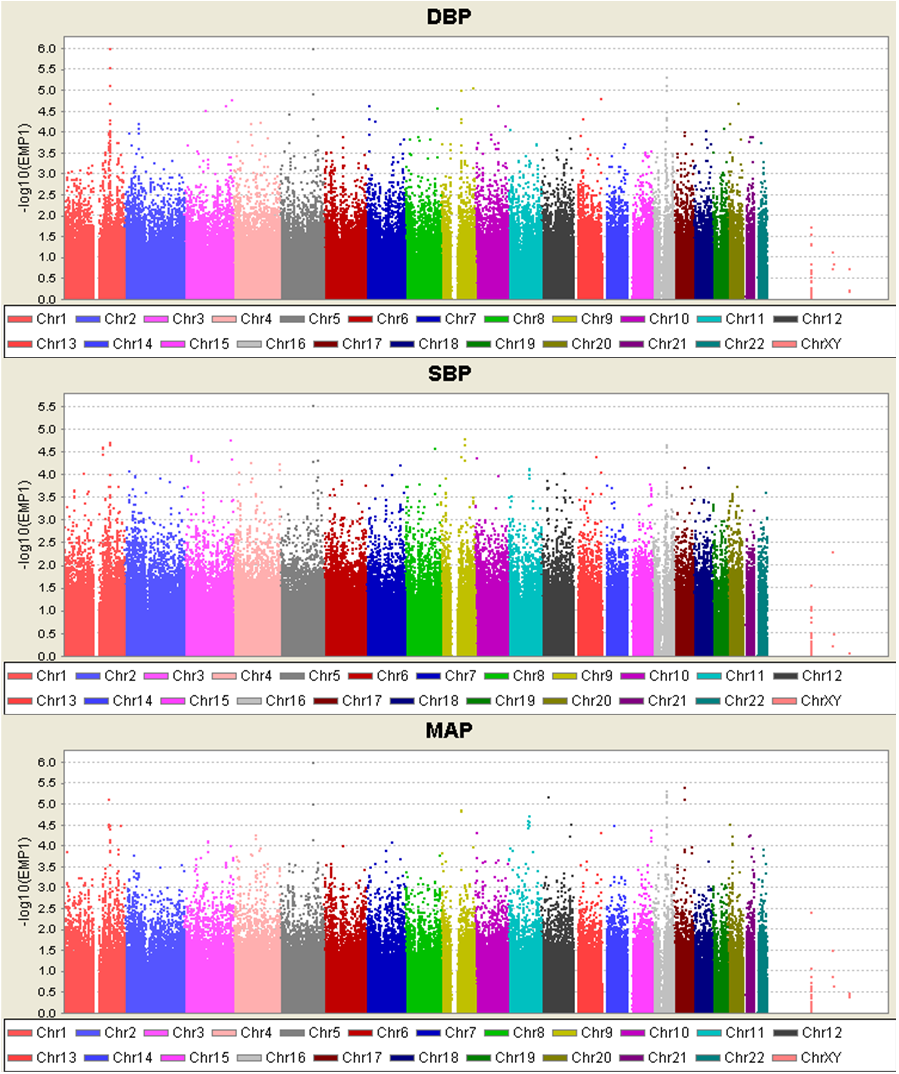

Supplement: Figure S2 — Plots of whole genome association scan results for three quantitative traits using within-family tests. SNPs from each chromosome are represented by a different color and ordered by physical location. (TIF) [file pone.0031489.s002.tif]

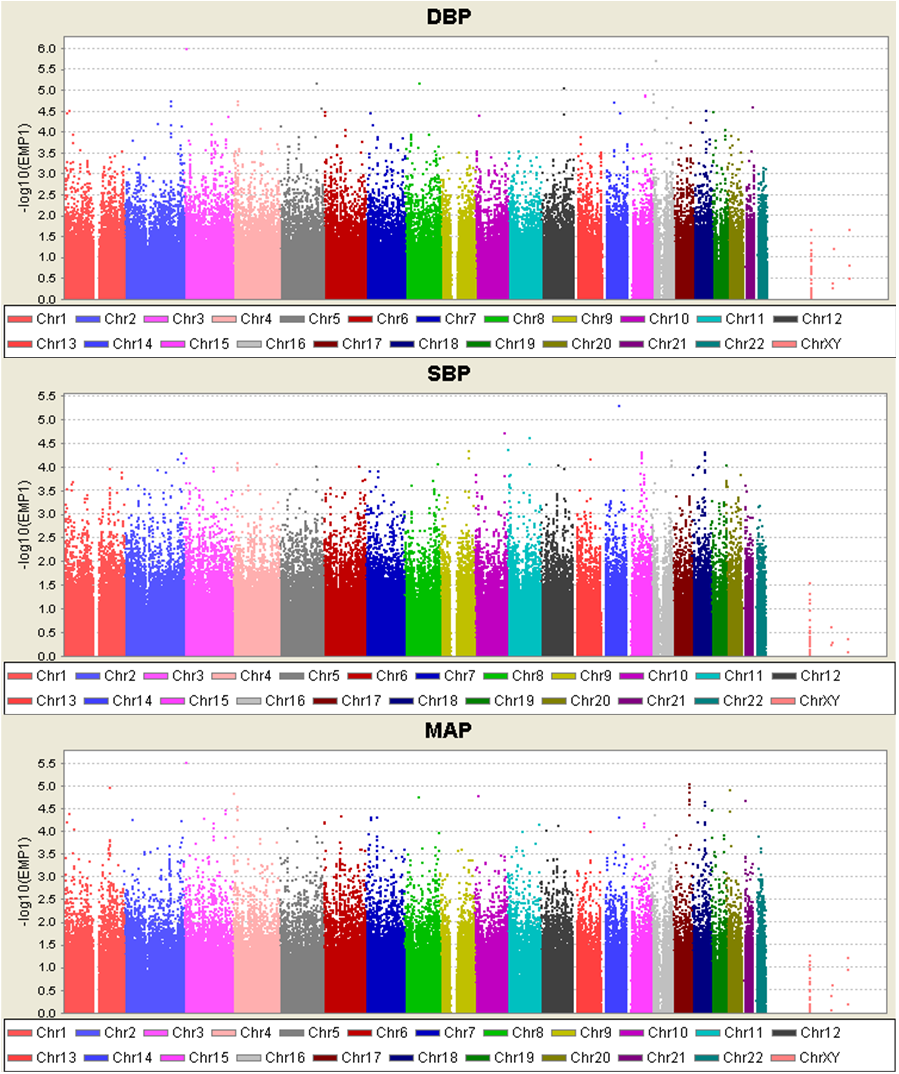

Supplement: Figure S3 — Plots of whole genome association scan results for three quantitative traits using the total association test. SNPs from each chromosome are represented by a different color and ordered by physical location. (TIF) [file pone.0031489.s003.tif]

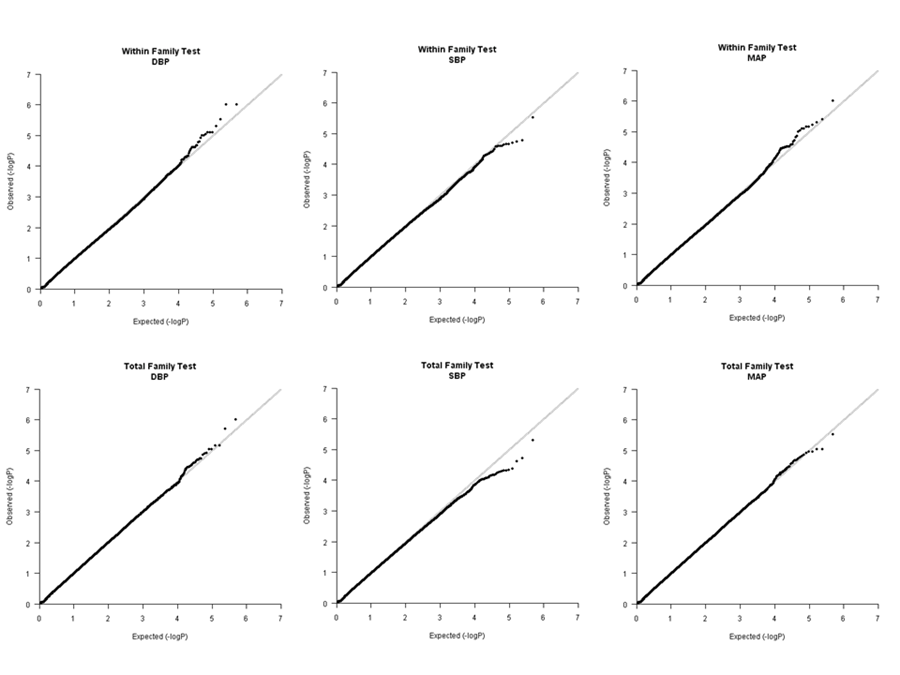

Supplement: Figure S4 — QQ plots of P -values observed vs expected under the null hypothesis, for the three quantitative traits (DBP, SBP and MAP), obtained from tests of within-family and total association. (TIF) [file pone.0031489.s004.tif]

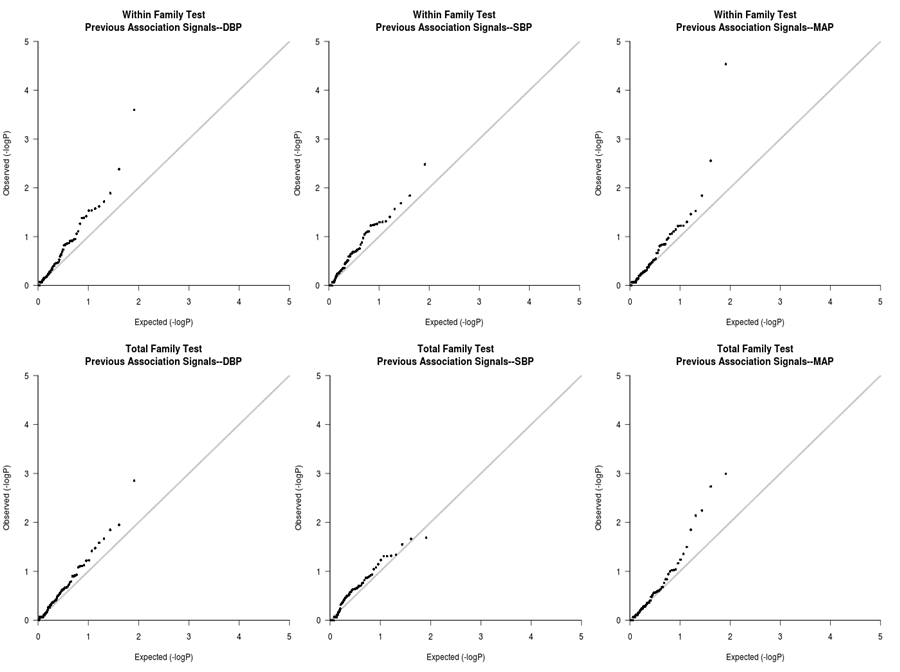

Supplement: Figure S5 — QQ plots for previous reported SNPs by eight genome-wide scan studies as well as candidate gene studies in our association analysis of within-family and total tests for DBP, SBP and MAP. (TIF) [file pone.0031489.s005.tif]

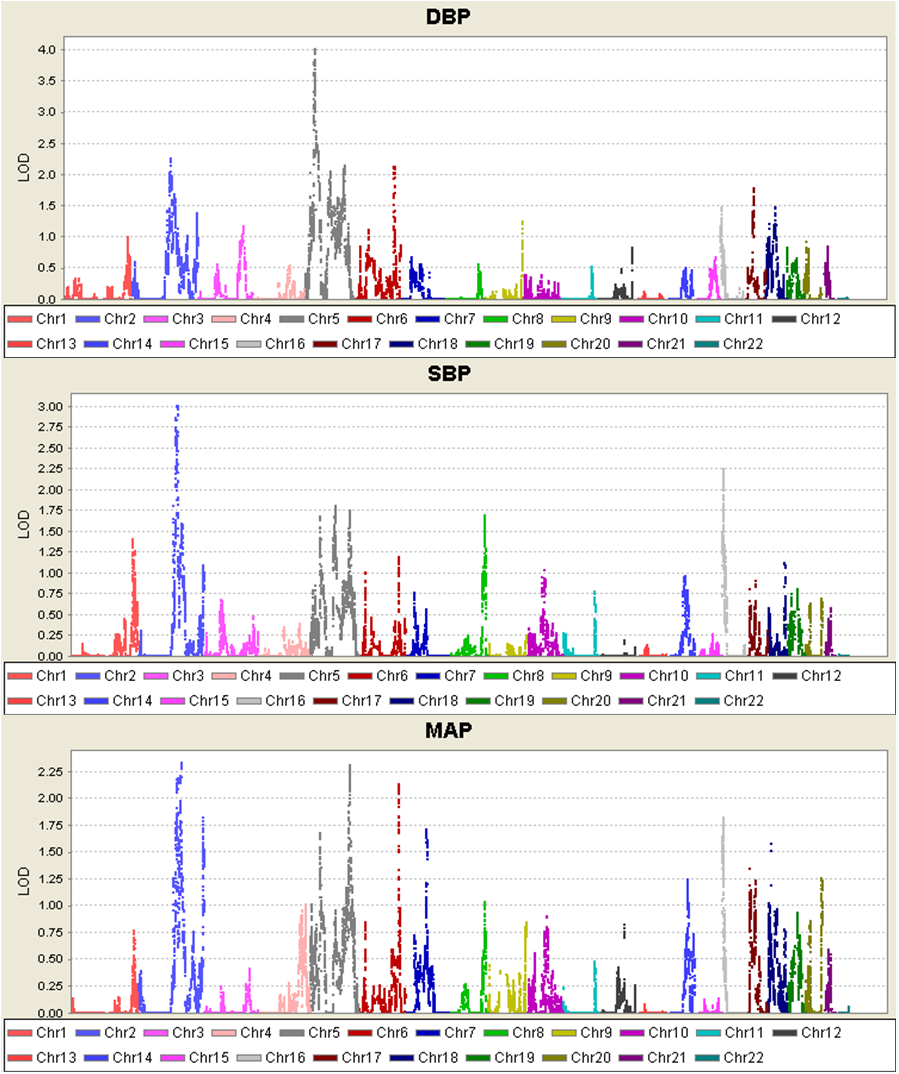

Supplement: Figure S6 — Results of the genome-wide linkage analysis are illustrated for DBP, SBP and MAP, respectively. The multipoint LOD scores are shown on the y-axis plotted against the chromosomal position on the x-axis. (TIF) [file pone.0031489.s006.tif]

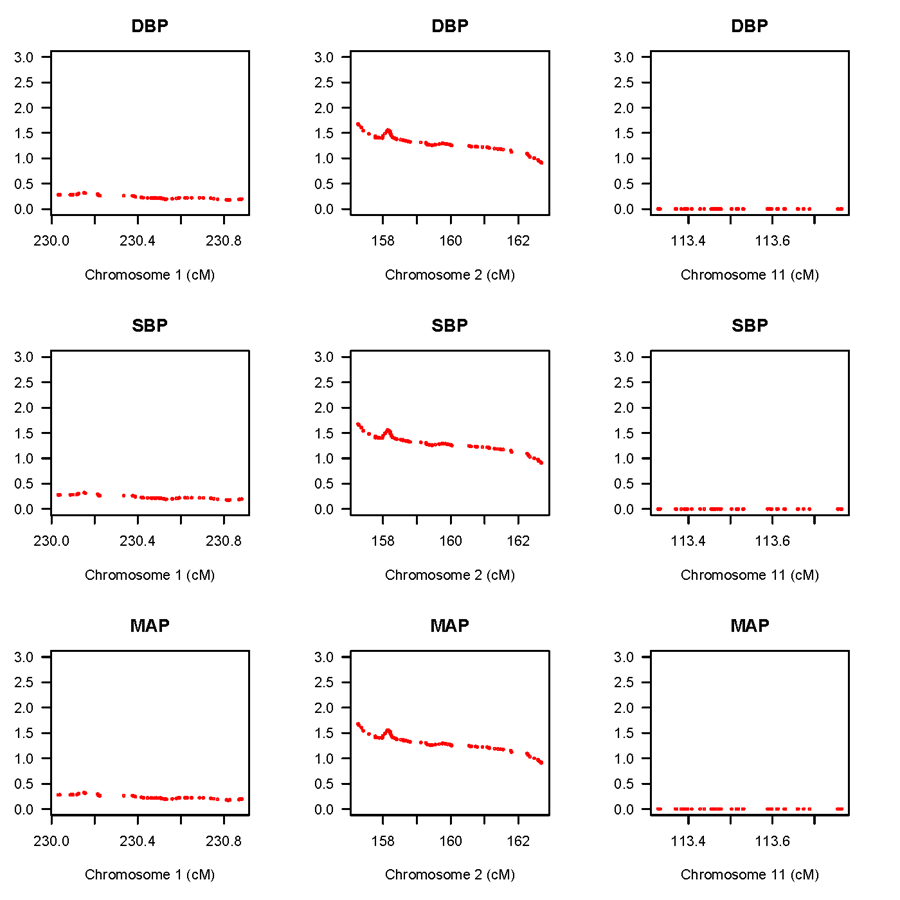

Supplement: Figure S7 — Three regions ( AGT , 2q14-q23 and DRD2 ) with evidence of linkage are shown in the results of linkage analysis as illustrated for DBP, SBP and MAP, respectively. The multipoint LOD scores are shown on the y-axis plotted against the chromosomal position on the x-axis. (TIF) [file pone.0031489.s007.tif]

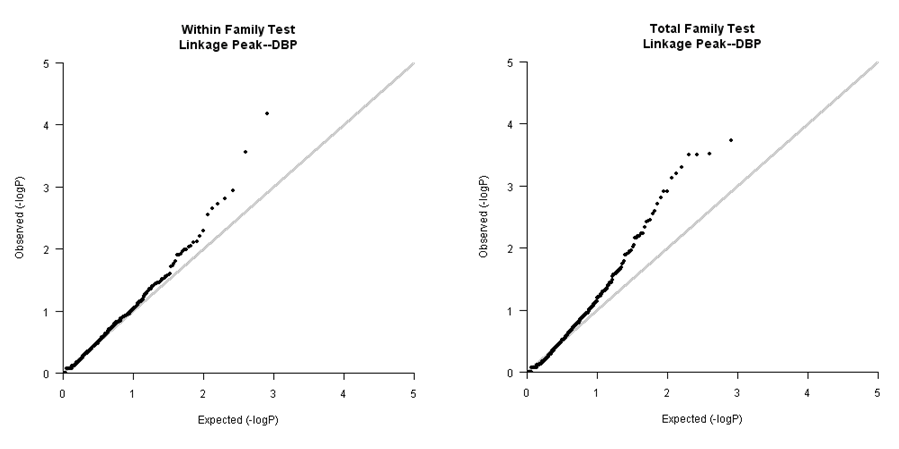

Supplement: Figure S8 — QQ plots of the Merlin-Regress peak findings in within-family association analysis for DBP. (TIF) [file pone.0031489.s008.tif]
