# Supplementary material for: A Genome-Wide Linkage and Association Scan Reveals Novel Loci for Hypertension and Blood Pressure Traits
Source: PLoS One. 2012 Feb 24;7(2):e31489. doi: 10.1371/journal.pone.0031489 (PMC3286457; doi:10.1371/journal.pone.0031489)
Supplement: Table S1 — Strongest associations obtained for dichotomous hypertensive/normotensive disease status, sorted by P value. (PDF) [file pone.0031489.s009.pdf]

Table S1. Strongest associations obtained for dichotomous hypertensive/normotensive disease status, sorted by P value

SNP	Chr	Position	Minor allele	MAF	*P*
rs6596140	5	133049750	T	0.34	$8.97{\times}10^{-8}$
rs6596142	5	133049839	G	0.38	$5.95{\times}10^{-6}$
rs10021303	4	96075704	A	0.31	$7.44{\times}10^{-6}$
rs1110183	9	38446365	G	0.46	$8.48{\times}10^{-6}$
rs10973834	9	38447824	C	0.46	$1.01{\times}10^{-5}$
rs8086733	18	27901867	G	0.31	$1.06{\times}10^{-5}$
rs4664076	2	152629737	G	0.19	$2.19{\times}10^{-5}$
rs1950918	14	51049143	A	0.33	$2.31{\times}10^{-5}$
rs1510795	4	91720637	T	0.43	$2.95{\times}10^{-5}$
rs1381330	11	98708660	G	0.2	$3.07{\times}10^{-5}$
