# Supplementary material for: A Genome-Wide Linkage and Association Scan Reveals Novel Loci for Hypertension and Blood Pressure Traits
Source: PLoS One. 2012 Feb 24;7(2):e31489. doi: 10.1371/journal.pone.0031489 (PMC3286457; doi:10.1371/journal.pone.0031489)
Supplement: Table S2 — Strongest associations obtained from both within and total tests for DBP, SBP and MAP, sorted by P value. (PDF) [file pone.0031489.s010.pdf]

Table S2. Strongest associations obtained from both within and total tests for DBP, SBP and MAP, sorted by P value

	Within family test					Total family test				
Trait	SNP	Chr	Minor allele	Beta mmHg	*P*	SNP	Chr	Minor allele	Beta mmHg	*P*
DBP	rs4463623	1	C	8.93	1.00×10^{-6}	rs4686340	3	C	-10.14	1.00×10^{-6}
	rs6596140	5	C	-9.77	1.00×10^{-6}	rs2075514	16	T	5.78	2.00×10^{-6}
	rs4434808	1	C	8.93	3.00×10^{-6}	rs9325113	5	C	7.80	7.00×10^{-6}
	rs12930697	16	A	-6.96	5.00×10^{-6}	rs16921974	8	T	-11.88	7.00×10^{-6}
	rs1387343	1	T	-7.73	8.00×10^{-6}	rs7294593	12	G	5.11	9.00×10^{-6}
	rs11644632	16	G	-6.96	8.00×10^{-6}	rs7964689	12	T	5.11	9.00×10^{-6}
	rs1550823	16	G	-6.96	8.00×10^{-6}	rs8049367	16	C	-4.78	1.20×10^{-5}
	rs3012719	9	C	10.89	9.00×10^{-6}	rs11574484	15	T	6.53	1.30×10^{-5}
	rs1410542	9	T	8.87	1.00×10^{-5}	rs1044581	15	T	6.53	1.40×10^{-5}
	rs1037109	16	C	-6.96	1.00×10^{-5}	rs1899025	2	A	-5.02	1.80×10^{-5}
SBP	rs6596140	5	C	-11.97	3.00×10^{-6}	rs887506	14	C	-11.42	5.00×10^{-6}
	rs10981664	9	C	-13.87	1.70×10^{-5}	rs10886918	10	G	-6.13	1.90×10^{-5}
	rs6800495	3	G	11.19	1.80×10^{-5}	rs10830452	11	A	6.01	2.40×10^{-5}
	rs4463623	1	C	10.68	2.00×10^{-5}	rs11026055	11	G	8.15	4.32×10^{-5}
	rs4434808	1	C	10.68	2.20×10^{-5}	rs4574921	9	T	-6.33	4.71×10^{-5}
	rs11644632	16	G	-8.89	2.20×10^{-5}	rs12333	15	C	-10.84	4.98×10^{-5}
	rs2401383	9	C	-13.87	2.30×10^{-5}	rs9955776	18	G	-6.17	4.98×10^{-5}
	rs2147445	1	C	-12.12	2.60×10^{-5}	rs4530357	2	C	-5.93	5.04×10^{-5}
	rs12930697	16	A	-8.89	2.60×10^{-5}	rs2456015	15	A	-10.84	5.28×10^{-5}
	rs1550823	16	G	-8.89	2.60×10^{-5}	rs4941229	18	T	-5.83	5.60×10^{-5}
MAP	rs6596140	5	C	-9.24	1.00×10^{-6}	rs4686340	3	C	-10.86	3.00×10^{-6}
	rs1867087	17	T	9.16	4.00×10^{-6}	rs2714017	17	C	-5.41	9.00×10^{-6}
	rs11644632	16	G	-7.24	5.00×10^{-6}	rs2713989	17	T	-5.37	9.00×10^{-6}
	rs12930697	16	A	-7.24	6.00×10^{-6}	rs284113	1	T	-5.04	1.10×10^{-5}
	rs16933533	12	C	7.95	7.00×10^{-6}	rs12948247	17	T	-5.41	1.10×10^{-5}
	rs1550823	16	G	-7.24	7.00×10^{-6}	rs737006	20	A	-19.70	1.20×10^{-5}
	rs6665157	1	G	6.92	8.00×10^{-6}	rs2714016	17	C	-5.41	1.40×10^{-5}
	rs4987082	17	A	-8.63	8.00×10^{-6}	rs4689408	4	C	-5.82	1.50×10^{-5}
	rs1037109	16	C	-7.24	9.00×10^{-6}	rs2296596	10	A	-6.95	1.70×10^{-5}
	rs6596142	5	A	-8.93	1.00×10^{-5}	rs16921974	8	T	-13.02	1.80×10^{-5}

Shown is the top 10 SNPs for each independent locus associated with systolic blood pressure, diastolic blood pressure and mean arterial pressure in within- and total- family tests of association. Effect sizes are on the mm Hg scale for increasing copy of the minor allele as estimated
