# Supplementary material for: A Genome-Wide Linkage and Association Scan Reveals Novel Loci for Hypertension and Blood Pressure Traits
Source: PLoS One. 2012 Feb 24;7(2):e31489. doi: 10.1371/journal.pone.0031489 (PMC3286457; doi:10.1371/journal.pone.0031489)
Supplement: Table S3 — Relationship of SNPs at 9 significant loci to three blood pressure traits. (PDF) [file pone.0031489.s011.pdf]

Table S3. Relationship of SNPs at 9 significant loci to three blood pressure traits

SNP ID	Chr	Position	Minor allele	MAF	Trait	Within-family test		Total-family test	
						Beta	*P*	Beta	*P*
rs4463623	1	186567491	C	0.465	**DBP**	8.93	1.00×10^{-6}	2.98	5.42×10^{-3}
					SBP	10.68	2.00×10^{-5}	5.07	6.02×10^{-4}
					MAP	7.97	3.40×10^{-5}	4.28	2.13×10^{-4}
rs4434808	1	186574912	C	0.465	**DBP**	8.93	3.00×10^{-6}	2.98	6.51×10^{-3}
					SBP	10.68	2.20×10^{-5}	5.07	7.16×10^{-4}
					MAP	7.97	4.00×10^{-5}	4.28	1.96×10^{-4}
rs1387343	1	186595273	T	0.473	**DBP**	-7.73	8.00×10^{-6}	-2.72	7.89×10^{-3}
					SBP	-8.80	3.11×10^{-4}	-4.34	2.47×10^{-3}
					MAP	-7.13	1.38×10^{-4}	-4.22	1.60×10^{-4}
rs6596140	5	133049750	C	0.337	**DBP**	-9.77	1.00×10^{-6}	-2.91	7.55×10^{-3}
					SBP	-11.97	3.00×10^{-6}	-2.74	7.85×10^{-2}
					MAP	-9.24	1.00×10^{-6}	-2.02	8.00×10^{-2}
rs6596142	5	133049839	A	0.378	**DBP**	-9.21	1.20×10^{-5}	-2.59	2.01×10^{-2}
					SBP	-10.76	5.27×10^{-5}	-2.74	1.03×10^{-1}
					MAP	-8.93	1.00×10^{-5}	-1.75	1.50×10^{-1}
rs9325113	5	148078196	C	0.086	**DBP**	8.13	1.57×10^{-3}	7.80	7.00×10^{-6}
					SBP	12.71	1.37×10^{-4}	9.40	9.68×10^{-5}
					MAP	9.58	1.16×10^{-3}	7.57	1.26×10^{-4}
rs2075514	16	15780110	T	0.294	**DBP**	6.43	3.97×10^{-3}	5.78	2.00×10^{-6}

					SBP	6.14	3.03×10^{-2}	6.22	2.16×10^{-4}
					MAP	5.49	1.65×10^{-2}	5.44	4.47×10^{-5}
rs12930697	16	60033003	A	0.481	**DBP**	-6.96	5.00×10^{-6}	-2.01	3.38×10^{-2}
					SBP	-8.89	2.60×10^{-5}	-2.53	7.17×10^{-2}
					MAP	-7.24	6.00×10^{-6}	-2.48	1.95×10^{-2}
rs1550823	16	60040317	G	0.476	**DBP**	-6.96	8.00×10^{-6}	-2.02	4.92×10^{-2}
					SBP	-8.89	2.60×10^{-5}	-2.57	7.72×10^{-2}
					MAP	-7.24	7.00×10^{-6}	-2.47	2.28×10^{-2}

MAF, minor allele frequency. Beta is the effect size per allele on blood pressure in mm Hg.
